# Evaluation of Photoluminescence Properties of Some Poly(ethylene glycol) – Supported Coumarin Derivatives

**DOI:** 10.3390/molecules14031044

**Published:** 2009-03-05

**Authors:** Graziella Tocco, Carlo Maria Carbonaro, Gabriele Meli, Gianni Podda

**Affiliations:** 1 Dipartimento Farmaco Chimico Tecnologico, Università degli Studi di Cagliari, Via Ospedale 72, 09124 Cagliari, CA, Italy; E-mails: cagi1978@gmail.com (G.M.), gpodda@unica.it (G.P.); 2 Dipartimento di Fisica- Università degli Studi di Cagliari, S.P. n°8- 09042 Monserrato (Cagliari), Italy

**Keywords:** Poly(ethylene glycol), Photoluminescence, Coumarins, PEG-conjugation.

## Abstract

The immobilization of some coumarin derivatives on modified poly(ethylene glycol)s is reported and the influence of the polymeric support on the photoluminescence activity of the compounds is discussed. Upon ultraviolet excitation, the derivatives showed coumarin - related emission properties whose peak position and efficiency depended on the loading of the polymer and on the mesomeric effects of the substituents.

## Introduction

Growing interest in coumarin chemistry was sparked by the discovery of their versatility in a large number of applications. Over the last few decades, in fact, several polycyclic compounds containing at least one coumarinic unit have been isolated from natural products and a lot of derivatives have been synthesized and investigated especially for their biological (anti-HIV, antibacterial, anti-hyperproliferative, anticoagulant) [1,2] and photophysical (as fluorescent tags and fluoroprobes) [3,4,5] properties. 

Few examples of coumarins supported on polymers were previously reported in the literature, with the aim of investigating their photophysical characteristics such as the photodimerization and the photocleveage of the coumarinic nucleus [5] or in an attempt to harvest and transfer solar radiation energy [4,6]. The photodimerization effect was successfully used to reversibly control the release of guest molecules trapped within coumarin modified mesoporous silica [8]. Besides, due to its major applications in the optoelectronic field as UV-blue emitters, silylated coumarin dyes [9] and coumarin-containing poly(fluorenediylvinylene)s [10] were synthesized to obtain a solid state and in solution polymer-light emitting devices.

In this respect, the immobilization of the coumarinic nucleus on water-soluble supports could represent a promising approach, especially useful in optoelectronic applications. Consequently, we report herein the immobilization of some coumarin derivatives on modified poly(ethyleneglycol)s of suitable molecular weight by using ether bond anchoring, with the aim of avoiding coumarin solubility problems and an investigation of their photoluminescence properties, which are fundamental aspects in pharmaceutical applications [11].

## Results and Discussion

An important restriction to the clinical use of some coumarin derivatives is related to their poor water solubility which, sometimes, has hampered further testing and development [12]. In an attempt to solve these problems, we have recently synthesized 4-methylcoumarin derivatives analogous to compounds **6**, **9**, **14** and **17**, by using, for the first time, the soluble support approach applied to the von Pechmann reaction [13]. In the same way, we succeeded in the preparation of some other PEG-coumarins, based on naturally occurring phenols [14].

We focused our attention on poly(ethylene glycol)s [(PEG)s] since they are non-toxic, inexpensive, generally soluble in a wide variety of solvents, commercially available and easy to functionalize, also offering many advantages over non-soluble polymeric supports. Moreover, when the molecular weight of the support is ≥ 2,000, the PEG - supported molecule system can be readily isolated as a pure product by precipitation /filtration, without using any other purification technique [15].

Due to these features, PEG chemistry has shown broad based applications, which may be in large part ascribed to the use of PEG-conjugates to deliver drugs, oligonucleotides or enzymes [16]. It is well known, for example, that the use of coumarins in the phototherapy of many skin diseases, is due to their ability to undergo electronic transitions when UVA-irradiated, working as photosensitizer drugs [17,18]. Thus, the maintenance of the optical properties of the polymer-supported coumarin is a mandatory task which can be successfully investigated by UV fluorescence spectroscopy [19,20,21,22,23,24,25,26,27]. 

**Scheme 1:**
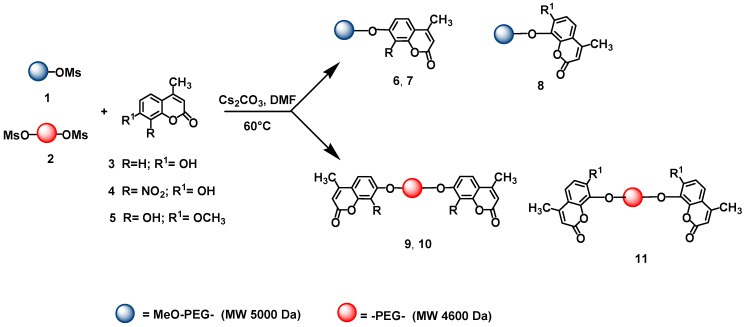
Immobilization of coumarins **3**, **4** and **5**.

In the research presented herein, the commercially available coumarins 7-hydroxy-4-methyl- coumarin (**3**), 7-hydroxy-4-methyl-8-nitrocoumarin (**4**) and 7-hydroxy-8- methoxy-4-methylcoumarin (**5**) have been immobilized on the previously mesylated polymeric supports **1** and **2** (Scheme 1). 

**Scheme 2:**
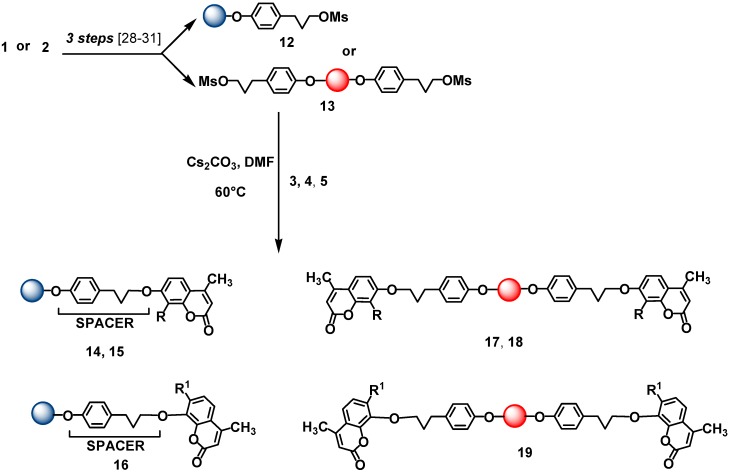
Immobilization of coumarins **3**, **4** and **5** in the presence of a spacer.

In some derivatives we inserted a spacer [28,29,30,31] between the support and the coumarin to test its possible influence on the photoluminescence properties of the anchored coumarin (Scheme 2). 

**Figure 1:**
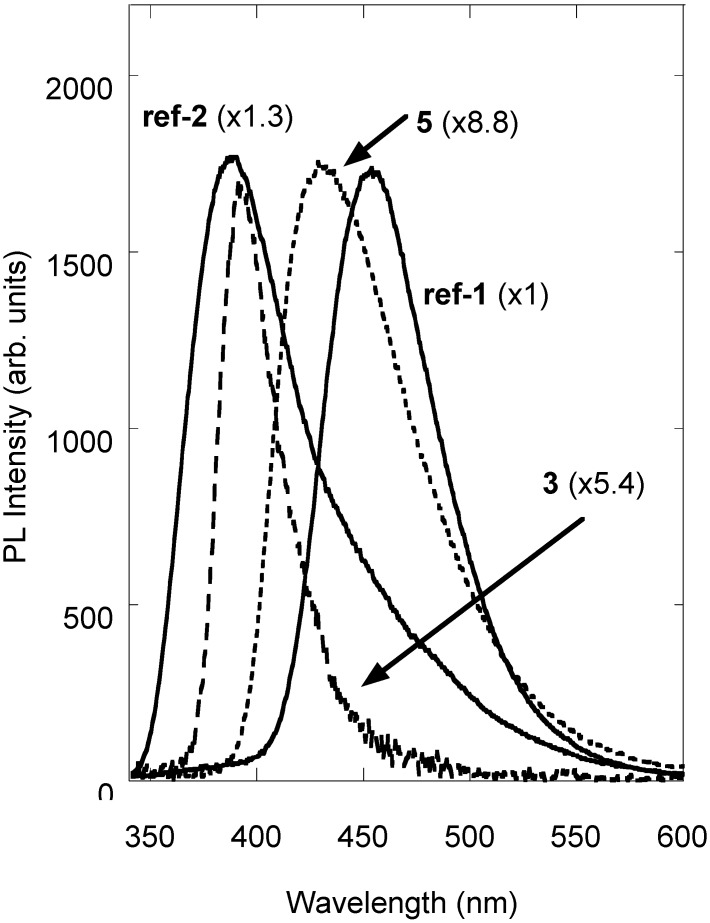
PL spectra of references (**ref-1** and **ref-2**) and coumarin samples (**3** and **5**).

Photoluminescence (PL) experiments have been carried out on all the synthesized compounds, investigating their emission properties by exciting the solid samples at 337 nm, because it is well known that 7-hydroxy-4-methylcoumarin displays an absorption band in the 300-400 nm range peaking at about 320 nm and PL features in the near UV-visible region [32]. The PL spectra of PEG-coumarins **3** and **5**, excited at 337 nm, together with the spectrum of a standard reference (a 5 x 10^-4^ M solution of 7-diethylamino-4-methylcoumarin in methanol, QE = 0.73, **ref-1**) are reported in Figure 1, while in Table 1 peak positions, full width at half maximum (fwhm) and quantum efficiency (QE) are listed. As shown, coumarins’ PL spectra display different features; in fact, we have a peak at 392 nm with a fwhm of 33 nm for compound **3**, while compound **5** shows a peak at 425 nm and a fwhm of 80 nm. The PL spectrum of **4** is not reported because no PL signal was detected under the selected excitation wavelength.

As a general rule, the emission properties of coumarins depend on the environmental conditions [9,32,33]. In particular, the luminescence of the 7-hydroxy-4-methylcoumarin (**3**) is centred at about 385 nm in neutral and slightly acidic solution and it is associated to the neutral molecule. On the contrary, the PL peak shows a bathochromic shift at about 455 nm in basic environments because of the anionic structure of the compound. Otherwise, under acidic conditions and in the presence of water, the zwitterionic exciplex emits at about 480 nm and in strongly acidic solutions the emission peak of the cationic form is at about 420 nm [9,32]. To a better understanding of the reported features, the PL spectrum of a methanol 7x10^-4^ M solution of **3** is reported in Figure 1 (**ref-2**). 

The spectrum of **ref-2** can be interpreted as the combination of the neutral and anionic ground state emissions. By comparison the PL band detected for **3** can be related to the neutral molecule. On the contrary, no similar characterization of the fluorescence for **5** was found in literature so that we could not verify the possible contributions of different chemical forms of **5** to the PL feature. The expected red-shift of the fluorescence of **5** compared to **3**, is associated to the presence of a strong electron-donor methoxyl group in 7-position, whose effect is improved by the mesomeric effect of the hydroxyl group in 8-position [32,34].

As compared to quantum efficiency of the references (Table 1), the powder samples have a reduced quantum yield due to the concentration quenching effect typically observed in highly concentrated solution or solid state samples. In addition, it has been previously reported that the concentration effect can cause a red-shift of the emissions [35]. Thus, it can be hypothesized a concentration effect in the double-loaded samples. Conversely, the anchoring procedure does not affect the emission efficiency of PEG-supported coumarins since their QE is comparable to the QE of the parent coumarin.

**Table 1:** Spectral characteristics of references and coumarin derivatives excited at 337 nm.

SAMPLE	λ_em_ (nm)	fwhm (nm)	QE (%)
**ref-1**	453	62	70
**ref-2**	388	57	55
**3**	392	33	13
**5**	430	74	8
**6**	397	82	11
**8**	435	150	0.4
**9**	442	60	17
**11**	441	130	0.6
**14**	396	74	14
**16**	463	240	0.2
**17**	447	64	8
**19**	488	160	0.1

**Figure 2:**
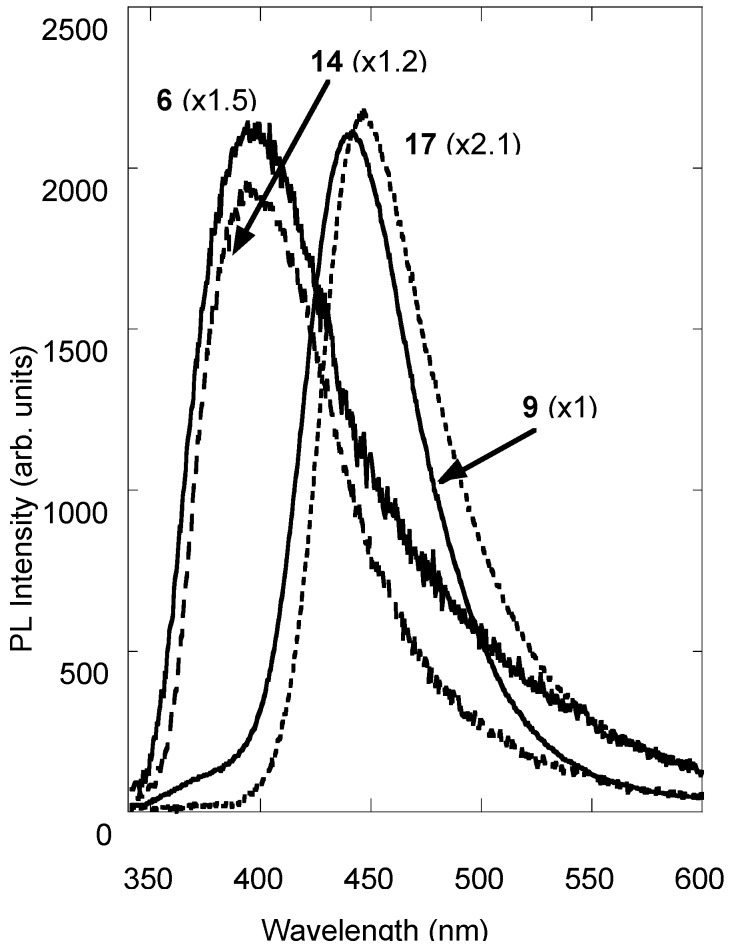
PL spectra of 7-hydroxy-4-methylcoumarin (**3**) derivatives (**6**, **9**, **14** and **17**).

Moreover, spectra and data reported in Figure 2 and in Table 1, show that **6** and **14** single-loaded derivatives present broadened and slightly red-shifted PL bands with respect to the emission features of the parent coumarin **3**. According to the attributions previously reported, **6** and **14** PL features can be assigned to the emission of the neutral and anionic form of the coumarin. A larger red shift is observed in the double-loaded derivatives **9** and **17** which can be ascribed to the concentration effect.

**Figure 3:**
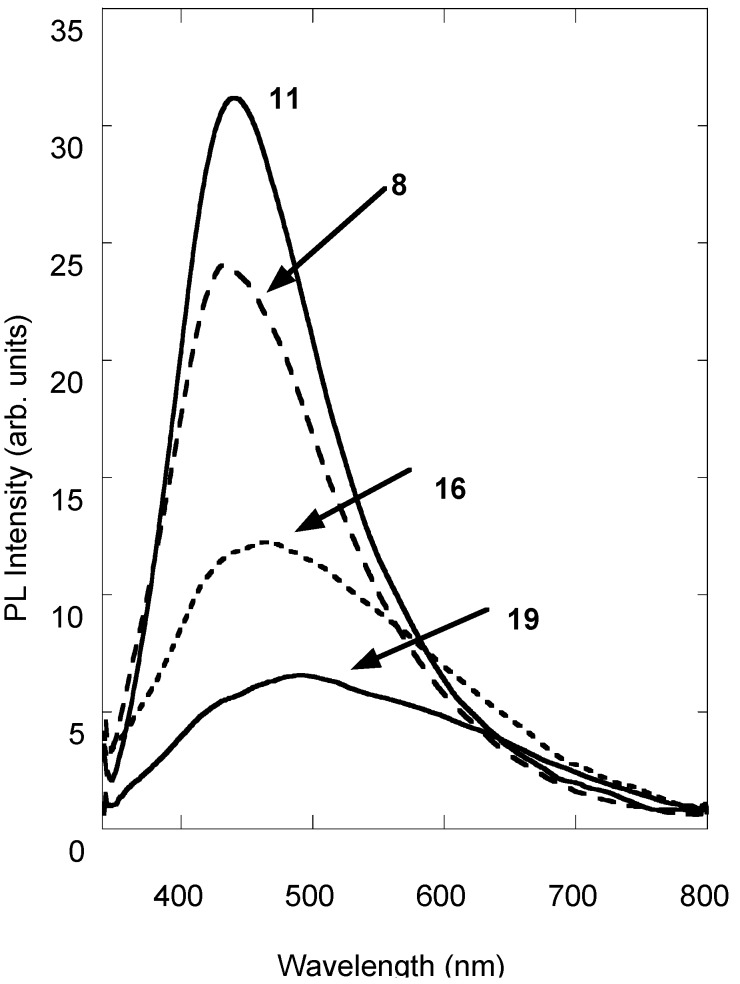
PL spectra of 8-hydroxy-7-methoxy-4-methylcoumarin derivatives (**8**, **11**, **16** and **19**).

PL spectra of compounds **8**, **11**, **16** and **19** are shown in Figure 3. As reported in Table 1, the peak position shifts bathocromatically up to 488 nm in compound **19**, whereas the fwhm is larger than that of the parent coumarin **5**, also with a greatly reduced emission efficiency. Both the effects could be explained by a different electronic distribution related to the presence of the O-PEG chain instead of the OH group in the 8-position. In these samples the electron-donor effect of the methoxyl group is the feature that mainly affects the electronic density distribution. In this respect, a red-shift of the absorption and emission bands should be expected and an increase of the detected QE should be observed by exciting the samples with a less energetic wavelength. 

Indeed, PL spectra recorded upon 365 nm excitation (Hg lamp) showed an enhancement of the efficiency (Table 2) and a red-shift of the emission band at about 485 nm.

**Table 2:** Spectral characteristics of **5** and **8**, **11**, **16** and **19** excited at 365 nm (RQE = relative quantum efficiency measured with respect to **5**).

SAMPLE	λ_em_ (nm)	RQE (%)
**5**	433	100
**8**	485	16
**11**	486	17
**16**	482	8
**19**	492	6

However, as previously discussed, the presence of different chemical forms of **5** with different spectral properties cannot be excluded. In addition, by comparing **3** and **5** derivatives, two different effects can be evidenced: 1) reduction of the emission efficiency compounds **8**, **11**, **16** and **19** with respect to the parent **5** coumarin (roughly one order of magnitude) whereas **3** derivatives show comparable QE; 2) no evident red-shift of PL emission of the double loaded derivatives **9** and **17** if compared to the single loaded **6** and **14** whereas a progressive red-shift of the PL in **5** derivatives is observed. Beyond the different electronic distribution, the anchoring of the PEG chain in the 8-position may also cause the excited coumarin to access different non-radiative decay paths leading to a less efficient emission and reducing, even completely, the extent of the concentration effect. In fact, the main photophysical features of coumarins can be interpreted as the emission from an intramolecular charge-transfer (ICT) excited state and a non-radiative decay related to the rotation of the emitting functionality at the 7-position. The latter mechanism, called twisted intramolecular CT (TICT), depends on the structure of the coumarin, the solvent polarity and the viscosity [33]. A detailed study of the absorption and emission properties of **5** as a function of the environmental conditions (i.e. pH of the solution and viscosity) is now under investigation. 

Concerning **4** and its derivatives **7**, **10**, **15** and **18**, no PL signal was recorded by exciting at 337 nm. In fact, the lack of emission under the applied experimental conditions was expected, since N-related groups usually cause a red-shift of the absorption and emission features of coumarin samples (R = NH_2_) and are responsible of quenching effects (R = NO_2_) [32,36]. Even by exciting at larger wavelength (the 365 nm line of Hg lamp) no PL signal was recorded. Further measurements are in progress to characterize the optical features of the selected nitro-coumarin and its derivatives.

A final comment pertains the photodimerization effect, which is typically observed in coumarin derivatives [34]. The laser power (of about 1 mW) and the irradiation time (few minutes) of the reported experiments are not sufficient to observe the cited effect. Indeed, the detected PL intensities do not decrease during the measurements, as expected if dimers were formed. 

Lastly, we have also verified that neither the support (PEG) nor the spacer contribute to the optical properties reported. As a consequence, the non UV-absorbing properties of our PEG-modified supports (at least at the excitation wavelength of 337.1 nm) could also represent an interesting option to carry out photochemical reactions under liquid phase conditions.

## Conclusions

In conclusion, we have succeeded in supporting some coumarin derivatives on PEG using an ether bond to anchor the coumarin onto the polymer, instead of the more often used ester linkage. Our aim, in fact, was to prepare a very stable system able to also resist drastic chemical or physical experimental conditions. The photoluminescence activity of the synthesized powders were investigated by exciting in the UV region. The resulting species still showed coumarin related photoluminescence properties, allowing a possible biological application of the supported systems.

As predicted, all the compounds are easily dissolvable in a wide variety of solvents, water included, more than the pure coumarins and this could be an important and intriguing feature for a pharmaceutical application. Moreover, the PEG-conjugation technique could represent a valid way to improve the biological properties of the linked molecules, since the *pegylated* - coumarin systems could probably act as prodrugs, so improving the pharmacokinetics of the biologically active anchored molecule. In particular, the investigation of the spacer influence on the pharmacological activity of the *pegylated-*coumarins could be of great interest, as recently discussed for many other PEG-drug conjugates [16,37].

## Experimental

### General

All PEG samples (Aldrich and Fluka) were melted under vacuum at 90 °C for about 45 min before use, to remove any trace of moisture. After reaction, the crude mixture was concentrated under vacuum to eliminate the solvent, added up by few mL of CH_2_Cl_2_ and then centrifuged to eliminate the excess of insoluble coumarin. The obtained solution was poured into Et_2_O (50 mL* per* g of polymer) and cooled at 0 °C. The resulting suspension was filtered through a sintered glass filter and the obtained solid was repeatedly washed on the filter with pure Et_2_O. All samples have been crystallized from isopropyl alcohol to eventually eliminate the excess of the polar reagents or the byproducts. It is well known, in fact, that PEGs, as a result to their helical structure, show a strong propensity to crystallize [38,39]. The yields of PEG-supported compounds were determined by weight, and their purity was confirmed by ^1^H-NMR analysis in CDCl_3_ performed by a Varian 300 MHz using tetramethylsilane as internal standard and with a pre-saturation of the methylene signals of the polymeric support at 3.60 ppm. In recording the NMR spectra, a relaxation delay of 6 sec and an acquisition time of 4 sec were used to ensure complete relaxation and accuracy of integration. Coumarin compounds **3**, **4** and **5** were commercially available (Aldrich, Lancaster).

PL measurements were carried out by exciting the samples with the 337.1 nm wavelength of a pulsed N_2_ laser (PRA Laser inc., mod. LN100C). The excitation pulse duration was 300 ps, the mean power was 1 mW and the repetition rate 60 Hz. The PL signal was gathered by a photonic multichannel spectral analyzer (Hamamatsu PMA-11) in the 300-800 nm spectral range with a spectral bandwidth of 1 nm. The reported spectra are recorded applying a short wavelength cutoff filter (WG345) and corrected for the optical transfer function of the system. A quantitative estimation of the quantum efficiency (QE) of the samples was obtained by comparison with a standard reference (**ref-1**) [32]. A 10% error on the estimated QE was allowed. Some derivatives were investigated also with the 365 nm line of a Hg lamp and recorded with a GG400 short wavelength cutoff filter. PL measurements were carried out in front face configuration in order to minimize reabsorption effects [40].

### General procedure to immobilize coumarins ***3***, ***4*** and ***5***

Compounds ***6***-***8*** and ***14***-***16***: To a solution of **1** (0.98 mmol, previously dried under vacuum) in dry DMF (15 mL), Cs_2_CO_3_ (2.94 mmol) and commercial **3**, **4** or **5** (2.94 mmol) were added and the reaction mixture was stirred at 60 °C in nitrogen atmosphere for 48 h. After reaction, the mixture was filtered and the filtrate evaporated to dryness. The obtained crude product, dissolved in a few mL of CH_2_Cl_2_, was centrifuged to eliminate the excess of insoluble **3**, **4** or **5**. Compounds **6**-**8** and **14**-**16** were obtained as pure products by using the precipitation-filtration technique (see General). 

Compounds **9-11 and 17-19**: These compounds were prepared from **2** (1.05 mmol), **3**, **4** or **5** (6.30 mmol) and Cs_2_CO_3 _(6.30 mmol) in dry DMF (20 mL), following the same procedure as for compounds **6**-**8** and **14**-**16**.

### Analytical details for compounds ***6*** – ***19***

Compound **6**: Yield: 85%; ^1^H-NMR ppm: 2.40 (s, 3H), 3.36 (s, 3H), 6.25 (s, 1H), 6.35 (s, 1H), 6.82 (d, ^3^J = 8.50 Hz, 1H), 7.60 (d, ^3^J = 8.50 Hz, 1H); Compound **7**: Yield: 79%; ^1^H-NMR ppm: 2.42 (s, 3H), 3.36 (s, 3H), 6.20 (s, 1H), 6.95 (d,^ 3^J = 8.20 Hz, 1H), 7.91 (d, ^3^J = 8.20 Hz, 1H); Compound **8:** Yield: 83%; ^1^H-NMR ppm 2.38 (s, 3H), 3.40 (s, 3H), 3.75 (s, 3H), 6.21 (s, 1H), 6.73 (d, ^3^J = 7.98 Hz, 1H), 7.41 (d, ^3^J = 7.98 Hz, 1H); Compound **14**: Yield: 77%; ^1^H-NMR ppm: 1.64 (m, 2H), 2.51 (s, 3H), 2.70 (t, ^3^J = 7.40 Hz, 2H), 3.36 (s, 3H), 3.92 (t, ^3^J = 6.70 Hz, 2H), 6.18 (s, 1H), 6.30 (s, 1H), 6.81 (m, 2H), 6.88 (m, 3H), 7.50 (d, ^3^J = 8.41, 1H); Compound **15**: Yield: 74%; ^1^H-NMR ppm: 1.77 (m, 2H), 2.40 (s, 3H), 2.67 (m, 2H), 3.36 (s, 3H), 4.12 (t,^ 3^J = 6.50 Hz, 2H), 6.18 (s, 1H), 6.72 (m, 2H), 6.94 (m, 3H), 7.88 (d, ^3^J = 8.17 Hz, 1H); Compound **16**: Yield: 75%; ^1^H-NMR ppm: 1.81 (m, 2H), 2.40 (s, 3H), 2.62 (t, ^3^J = 7.22 Hz, 2H), 3.36 (s, 3H), 3.75 (s, 3H), 4.20 (t, ^3^J = 6.70 Hz, 2H), 6.18 (s, 1H), 6.48 (d, ^3^J = 8.30 Hz, 2H), 6.73 (d, ^3^J = 8.85 Hz, 1H), 6.98 (d,^ 3^J = 8.30 Hz, 2H), 7.43 (d, ^3^J = 8.85 Hz, 1H); Compound **9**: Yield: 87%; ^1^H-NMR ppm: 2.45 (s, 6H), 6.21 (s, 2H), 6.48 (s, 2H), 6.90 (d, ^3^J = 8.45 Hz, 2H), 7.65 (d, ^3^J = 8.45 Hz, 2H); Compound **10**: Yield: 80%; ^1^H-NMR ppm: 2.51 (s, 6H), 6.38 (s, 2H), 7.02 (d,^ 3^J = 8.20 Hz, 2H), 7.84 (d, ^3^J = 8.20 Hz, 2H); Compound **11**: ^1^H-NMR ppm: 2.36 (s, 6H), 3.83 (s, 6H), 6.17 (s, 2H), 6.70 (d, ^3^J = 8.01 Hz, 2H), 7.51 (d, ^3^J = 8.01 Hz, 2H); Compound **17**: Yield: 76%; ^1^H-NMR ppm: 1.58 (m, 4H), 2.42 (s, 6H), 2.74 (t, ^3^J = 7.65 Hz, 4H), 4.00 (t, ^3^J = 6.81 Hz, 4H), 6.16 (s, 2H), 6.30 (s, 2H), 6.75 (m, 4H), 6.89 (m, 6H), 7.47 (d, ^3^J = 8.44, 2H); Compound **18**: Yield: 73%; ^1^H-NMR ppm: 1.89 (m, 4H), 2.44 (s, 6H), 2.60 (m, 4H), 4.25 (t,^ 3^J = 6.62 Hz, 4H), 6.14 (s, 2H), 6.68 (m, 4H), 7.02 (m, 6H), 7.94 (d, ^3^J = 8.11 Hz, 2H); Compound **19**: Yield: 75%; ^1^H-NMR ppm: 2.01 (m, 4H), 2.49 (s, 6H), 2.70 (t, ^3^J = 7.14 Hz, 4H), 3.71 (s, 6H), 4.15 (t, ^3^J = 6.74 Hz, 4H), 6.20 (s, 2H), 6.50 (d, ^3^J = 8.15 Hz, 4H), 6.75 (d, ^3^J = 8.90 Hz, 2H), 7.00 (d,^ 3^J = 8.15 Hz, 4H), 7.48 (d, ^3^J = 8.90 Hz, 2H).
